# Molecular and Microbial Microenvironments in Chronically Diseased Lungs Associated with Cystic Fibrosis

**DOI:** 10.1128/mSystems.00375-19

**Published:** 2019-09-24

**Authors:** Alexey V. Melnik, Yoshiki Vázquez-Baeza, Alexander A. Aksenov, Embriette Hyde, Andrew C. McAvoy, Mingxun Wang, Ricardo R. da Silva, Ivan Protsyuk, Jason V. Wu, Amina Bouslimani, Yan Wei Lim, Tal Luzzatto-Knaan, William Comstock, Robert A. Quinn, Richard Wong, Greg Humphrey, Gail Ackermann, Timothy Spivey, Sharon S. Brouha, Nuno Bandeira, Grace Y. Lin, Forest Rohwer, Douglas J. Conrad, Theodore Alexandrov, Rob Knight, Pieter C. Dorrestein, Neha Garg

**Affiliations:** a Collaborative Mass Spectrometry Innovation Center, Skaggs School of Pharmacy and Pharmaceutical Sciences, University of California, San Diego, La Jolla, California, USA; b Jacobs School of Engineering, University of California, San Diego, La Jolla, California, USA; c Department of Pediatrics, University of California, San Diego, La Jolla, California, USA; d School of Chemistry and Biochemistry, Georgia Institute of Technology, Atlanta, Georgia, USA; e Department of Computer Science & Engineering, University of California, San Diego, La Jolla, California, USA; f Structural and Computational Biology Unit, European Molecular Biology Laboratory, Heidelberg, Germany; g Biology Department, San Diego State University, San Diego, California, USA; h Department of Pathology, University of California, San Diego, La Jolla, California, USA; i Department of Radiology, University of California, San Diego, La Jolla, California, USA; j Department of Medicine, University of California, San Diego, La Jolla, California, USA; k UC San Diego Center for Microbiome Innovation, University of California, San Diego, La Jolla, California, USA; l Department of Bioengineering, University of California, San Diego, La Jolla, California, USA; m Emory-Children’s Center for Cystic Fibrosis and Airways Disease Research, Atlanta, Georgia, USA; n Center for Microbial Dynamics and Infection, Georgia Institute of Technology, Atlanta, Georgia, USA; University of Copenhagen

**Keywords:** GNPS, *Pseudomonas*, spatial mapping, *Stenotrophomonas*, antibiotic distribution, cystic fibrosis, metabolomics, microbiome

## Abstract

To visualize the personalized distributions of pathogens and chemical environments, including microbial metabolites, pharmaceuticals, and their metabolic products, within and between human lungs afflicted with cystic fibrosis (CF), we generated three-dimensional (3D) microbiome and metabolome maps of six explanted lungs from three cystic fibrosis patients. These 3D spatial maps revealed that the chemical environments differ between patients and within the lungs of each patient. Although the microbial ecosystems of the patients were defined by the dominant pathogen, their chemical diversity was not. Additionally, the chemical diversity between locales in the lungs of the same individual sometimes exceeded interindividual variation. Thus, the chemistry and microbiome of the explanted lungs appear to be not only personalized but also regiospecific. Previously undescribed analogs of microbial quinolones and antibiotic metabolites were also detected. Furthermore, mapping the chemical and microbial distributions allowed visualization of microbial community interactions, such as increased production of quorum sensing quinolones in locations where *Pseudomonas* was in contact with *Staphylococcus* and *Granulicatella*, consistent with *in vitro* observations of bacteria isolated from these patients. Visualization of microbe-metabolite associations within a host organ in early-stage CF disease in animal models will help elucidate the complex interplay between the presence of a given microbial structure, antibiotics, metabolism of antibiotics, microbial virulence factors, and host responses.

**IMPORTANCE** Microbial infections are now recognized to be polymicrobial and personalized in nature. Comprehensive analysis and understanding of the factors underlying the polymicrobial and personalized nature of infections remain limited, especially in the context of the host. By visualizing microbiomes and metabolomes of diseased human lungs, we reveal how different the chemical environments are between hosts that are dominated by the same pathogen and how community interactions shape the chemical environment or vice versa. We highlight that three-dimensional organ mapping methods represent hypothesis-building tools that allow us to design mechanistic studies aimed at addressing microbial responses to other microbes, the host, and pharmaceutical drugs.

## Abstract (precis)

Microbial infections are now recognized to be polymicrobial and personalized in nature. Comprehensive analysis and understanding of the factors underlying the polymicrobial and personalized nature of infections remain limited, especially in the context of the host. By visualizing microbiomes and metabolomes of diseased human lungs, we reveal how different the chemical environments are between hosts that are dominated by the same pathogen and how community interactions shape the chemical environment or vice versa. We highlight that three-dimensional organ mapping methods represent hypothesis-building tools that allow us to design mechanistic studies aimed at addressing microbial responses to other microbes, the host, and pharmaceutical drugs.

## INTRODUCTION

An increasing rate of infection from multidrug-resistant opportunistic pathogens has become a significant burden in recent years. Proliferation of these pathogens due to overuse of antibiotics, including antibiotics of last resort (1–4), is a threat to human health and is already associated with increased mortality (5, 6). One reason for indiscriminate use of broad-spectrum antibiotics and combination therapy in complex polymicrobial infections is the lack of knowledge with regard to how microorganisms interact with each other, the host, and their chemical environment, leading to strategies that target bacterial pathogens broadly. Thus, specific microbial pathways that are involved in detrimental microbe-microbe interactions (7), microbe-host interactions (8), and microbe-drug interactions (9) can serve as new targets for targeted drug discovery. Knowledge of such interaction-mediating microbial pathways and their prevalence will shape the future of drug discovery. In this regard, even though we have begun to appreciate the presence of multiple subpopulations by imaging of community structures (10–12) and by genome sequencing (13–15), information about the specific microbial pathways involved in mediating the interactions mentioned above, about the molecular distribution of xenobiotic compounds, and about how such distributions are associated with specific microbial structures within the context of a host is largely lacking.

We developed a methodology to map microbial and metabolite distributions in a human lung in three dimensions (3D) to identify pathways that may be mediating microbial interactions and to visualize the distribution of antibiotics in relation to microbial community structure (16). These three-dimensional organ maps allow visualization of chemical and microbial microenvironments and consequently may provide better insights into the complex processes that take place within a host. Here, we applied this methodology to elucidate spatial variation within and between the lungs of three individuals afflicted with cystic fibrosis (CF).

CF is a genetic disease caused by a mutation in the cystic fibrosis transmembrane conductance regulator (CFTR) gene that results in defects of the encoded CFTR protein. The primary function of CFTR protein is of an ion channel that regulates liquid volume (mucus) on epithelial cells through secretion of chloride ions and inhibition of sodium absorption. Due to defects in CFTR protein, sticky mucus accumulates in the upper airways and lungs of CF patients and serves as a growth medium for various microbes, including opportunistic pathogens, resulting in chronic and recurrent polymicrobial infections. In the 1930s, children diagnosed with CF died as infants shortly after diagnosis (17). Due to advances in modern medicine, including the use of antibiotics and better clinical management of the disease, individuals with CF can now expect to live on an average into their forties even though most patients are waitlisted for organ transplant by the time they reach adulthood (18). Improved clinical management is partly made possible by better understanding of the polymicrobial nature of the infections of the lung and development of antibiotic-based management of chronic infections targeting the polymicrobial community (19). However, the virulence of pathogens in microbial lung diseases such as CF, pneumonia, tuberculosis, and chronic obstructive pulmonary disease is mostly studied in cultures derived from pulmonary secretions, by genome sequencing, which does not represent complex *in vivo* conditions. Emergence of transcriptomics studies has revealed differences in phenotypes of pathogens in cultures and pathogens in clinical samples such as sputum and wound infections (20). Furthermore, failure in treating an infection in a complex organ such as a human lung may simply stem from the inability to treat localized infection foci, which can then spread to the entire organ or become systemic as in the case of infections caused by *Burkholderia*, for example (13, 14, 21, 22). Understanding how the production of microbial small molecules involved in pathogenicity and community interactions varies with lung biogeography, leading to infection hot spots, will enable the development of targeted antimicrobials and improved drug delivery vehicles (14, 21, 23). Thus, CF presents an important test case for improving strategies for management of polymicrobial infections, given better understanding of community structures and chemical environments within the host.

In this study, with the consent of the patients, we mapped the chemical and microbial makeup of six explanted lungs, removed during surgery from three CF patients, by using 3D volume cartography to understand how microbes, microbial molecules, and medications are distributed and metabolized throughout the organ, providing insights into microbe-microbe interactions.

## RESULTS AND DISCUSSION

The explanted lungs of three patients afflicted with CF were sectioned to inventory and map the associated microbiome and metabolome in three dimensions onto lung models built from computed tomography (CT) scans acquired prior to surgery (see Materials and Methods) (16). To perform 16S rRNA gene analysis, the tissue sections were swabbed, enabling detailed inventory of bacterial DNA present within the patients’ lungs. We refer to our analysis of 16S rRNA gene as inventory of the bacterial DNA and not of the bacteria themselves, since lungs associated with CF are known to contain a significant amount of DNA from dead cells as well as extracellular DNA (24). In total, six lungs from three patients contained bacteria that spanned 40 genera (see Table S1 in the supplemental material). Bar plots of the most frequently amplified genera and their relative abundances pooled for all anatomical locations are illustrated for each patient in Fig. S1a in the supplemental material. The relative abundances of these genera in individual sections of each patient are available in 3D maps (see below). The DNA of the most commonly occurring pathogenic organism in CF, Pseudomonas aeruginosa, was detected at highest frequency throughout the lungs of patients 1 and 3, whereas the lungs of patient 2 were dominated by DNA from the emerging pathogen *Stenotrophomonas*. Even though the microbial population within CF-associated lungs can be heterogeneous (25), dominance of a single pathogen in end-stage CF disease has been described extensively in previous studies (26–28).

10.1128/mSystems.00375-19.1 FIG S1 (a) Bar charts representing taxonomic summary of all samples for each patient are shown. The legend for the 9 most abundant taxa is shown. The complete taxonomic summary is available in Table S1. Complete descriptions of all OTUs for each of the individual sections are available as spatial maps corresponding to patient 1, patient 2, and patient 3. (b and c) The first and second principal components of a PCoA plot of weighted Unifrac distances corresponding to 16S rRNA data from three patients are shown. (d, e) The second and third principal components of a PCoA plot of weighted Unifrac distances corresponding to 16S rRNA data from three patients are shown. Download FIG S1, TIF file, 1.1 MB. Copyright © 2019 Melnik et al. 2019 Melnik et al. This content is distributed under the terms of the Creative Commons Attribution 4.0 International license.

10.1128/mSystems.00375-19.10 TABLE S1 Taxon summary table. Download Table S1, DOC file, 0.02 MB. Copyright © 2019 Melnik et al. 2019 Melnik et al. This content is distributed under the terms of the Creative Commons Attribution 4.0 International license.

**FIG 1:**
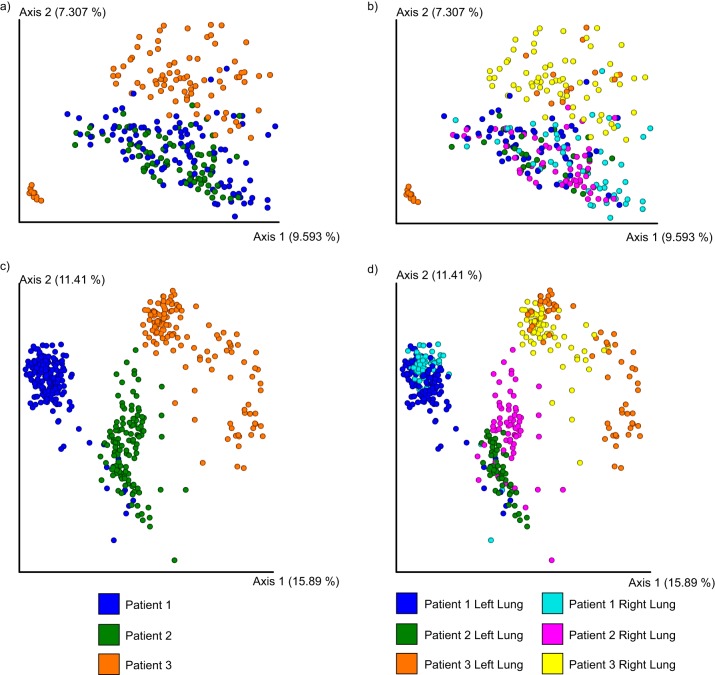
Principal-coordinate plot of metabolome and microbiome from lungs of three patients in the study. (a and b) PCoA plots of 16S rRNA sequencing with the unweighted UniFrac distance. (c and d) PCoA plots of the mass spectrometry data with Jaccard distance data.

The principal-component analysis (PCoA) of microbiome data with weighted UniFrac distance showed clustering between both lungs of patient 1 and the right lung of patient 3 along the first two principal axes (Fig. S1b). Samples from the left lung of patient 3 clustered separately, and comparisons of the 10 most abundant operational taxonomic units (OTUs) further highlighted the differences between the microbial communities present in the left and right lungs of patient 3 (Fig. S1c; see also Fig. S2a). Apart from the dominant pathogen, the overall microbiomes between and within patients were different along the second and third axes (see Fig. S1d and e). The unweighted UniFrac distance metric yielded a more homogeneous distribution of patients’ microbiome data in the PCoA space (Fig. 1a and b).

10.1128/mSystems.00375-19.2 FIG S2 (a) Top 10 most abundant OTUs and their ranks (1, most abundant; 10, least abundant) according to host and sampling site. Columns represent OTUs and are labeled according to their taxonomic classification and their Greengenes identifier. Rows represent the sites and are hierarchically clustered based on the rank vectors. (b) Weighted UniFrac distances from comparisons of patient 1 data to the data corresponding to the left and right lungs of patients 2 and 3. Download FIG S2, TIF file, 2.5 MB. Copyright © 2019 Melnik et al. 2019 Melnik et al. This content is distributed under the terms of the Creative Commons Attribution 4.0 International license.

**FIG 2:**
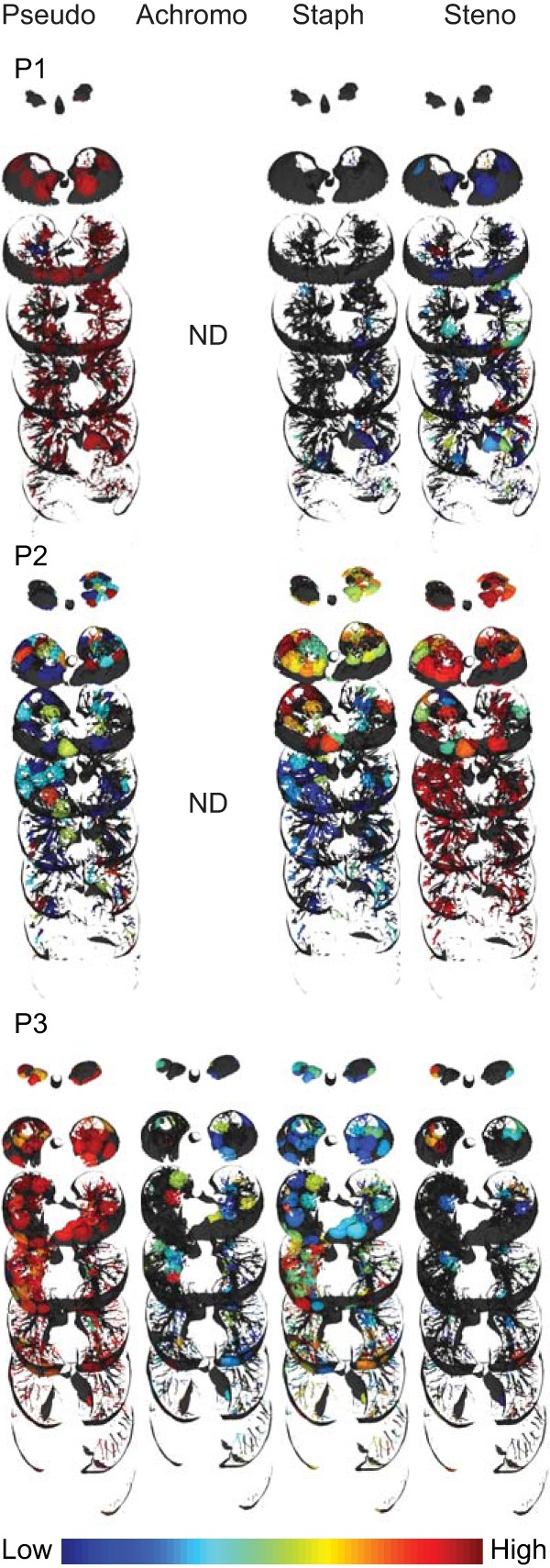
Distribution of microorganisms. Distributions of *Pseudomonas*, *Achromobacter*, *Staphylococcus*, and *Stenotrophomonas* (left to right) are shown for all three patients. Pseudo, *Pseudomonas*; Achromo, *Achromobacter*; Staph, *Staphylococcus*; Steno, *Stenotrophomonas*, P1, patient 1; P2, patient 2; P3, patient 3, ND, not detected. An intensity scale is provided at the bottom right. Full visualizations of microbial maps can be accessed via the following hyperlinks: patient 1, patient 2, and patient 3.

To map the relative frequencies of microbes onto the 3D lung models, we used our previously described methodology (16). The distribution of prevalent (*Pseudomonas* and *Staphylococcus*) and emerging (*Stenotrophomonas* and *Achromobacter*) microbes in the CF-associated lungs is displayed in Fig. 2. Although a nearly uniform distribution of dominant pathogens (*Pseudomonas* in patients 1 and 3 and *Stenotrophomonas* in patient 2) was observed, all other microbes were distributed unevenly, often being relegated to niche spots. For example, *Achromobacter* was mainly localized in the apex of the right lung of patient 3 whereas *Staphylococcus* was present in the lower lobe of both lungs of patient 1, at the apex of the lungs of patient 2, and in the middle and lower lobes of the lungs of patient 3. The dominant pathogen, *Stenotrophomonas*, showed uniform distribution in the lungs of the patient 2 and differential distributions in the lungs of patients 1 and 3 (Fig. 2). A degree of stratification is expected based on the availability of oxygen; *Achromobacter* and *Stenotrophomonas* are strict aerobes whereas *Staphylococcus* and *Pseudomonas* are facultative anaerobes residing as biofilms in airway mucus of CF patients with the potential of undergoing anaerobic metabolism (29). Furthermore, both lobes of patient 1 and the left lobe of patient 3 not only shared the dominant pathogen; their microbial communities were also more similar to each other than either was to that of patient 2 (Fig. S2a and b). Despite these similarities, comparing the communities by host based on permutational multivariate analysis of variance (PERMANOVA), we observe a strong personalized effect (*P* = 0.001; pseudo-*F* = 220.984). Selection pressures from competing microbes and chemical microenvironments, including antibiotic distributions, further leads to stratification of niches occupied by specific organisms. To compare the microbial and chemical environments, we next annotated the mass spectrometry (MS) data acquired from the tissue sections and mapped the data onto the 3D models of the lungs of these patients (see below).

**FIG 3:**
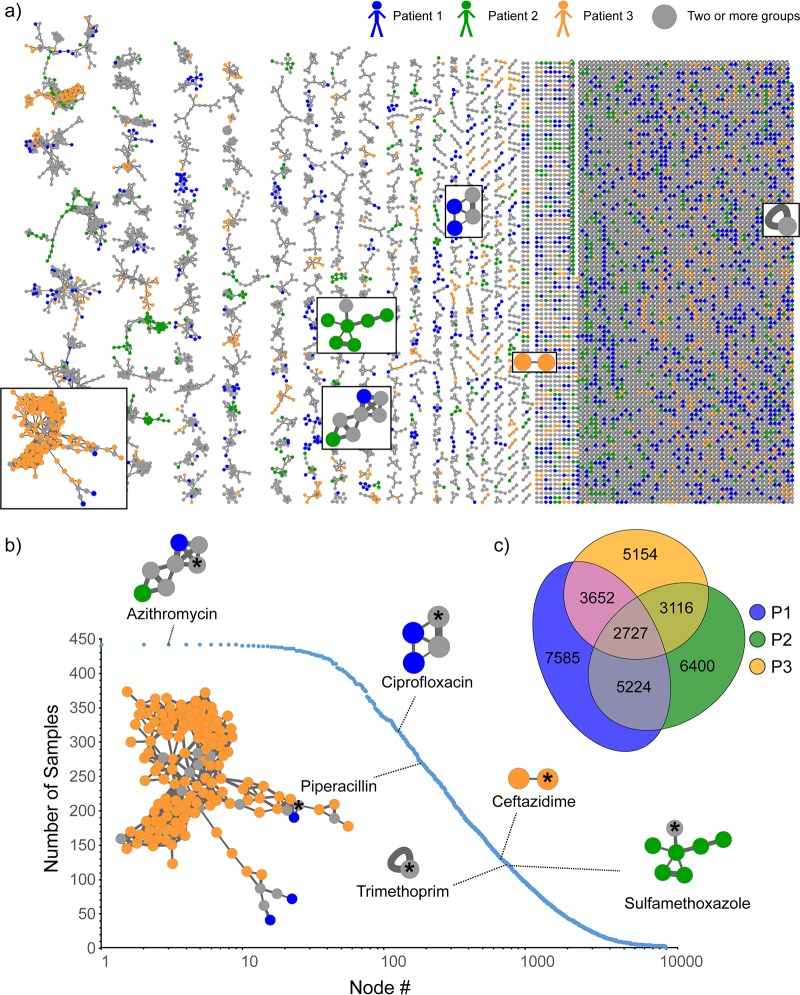
Molecular network analysis of all six lungs from three patients afflicted with CF. (a) The molecular network is color coded by patient as follows: blue, patient 1; green, patient 2; orange, patient 3. The network clusters corresponding to antibiotics are highlighted in boxes. (b) The numbers of samples that contained a given set of consensus MS/MS spectra (represented as nodes in panel a) are plotted. The frequency of occurrence of antibiotics detected in this data set is highlighted on the plot. The number of nodes in a cluster is reflective of the number of transformations of the parent compound that were detected. The node of each parent compound is highlighted an asterisk. The fragmentation patterns of the most frequently observed drugs, azithromycin and its analogs, are shown in Fig. S4; the large number of nodes shown in the piperacillin cluster stems from its structural similarity to small peptidic compounds abundant in biological samples and its inherent chemical reactivity with biological molecules (48). (c) Venn diagram of the overlap of consensus fragmentation spectra between three patients.

To annotate molecular ions detected using a high-resolution-MS-based untargeted approach, molecular network analysis was performed using the Global Natural Product Social Molecular Networking (GNPS) infrastructure (30). Molecular networking allows reduction and organization of the overwhelming amount of chemical information generated (in terms of mass spectra) in a high-resolution untargeted MS approach. The data reduction is performed by combining and displaying identical tandem MS (MS/MS) spectra as a single node and by displaying similar spectra as connected nodes (30, 31). Similarities in MS/MS spectra relate to similarities in chemical structures, so oftentimes such connected nodes represent chemical and biological transformations of a molecule. In this study, 676,451 MS/MS spectra were filtered and merged into consensus spectra, producing 9,874 nodes (Fig. 3a). The patient-specific molecules were displayed by assigning a specific color to the data from each patient in the molecular network analysis (Fig. 3a). In addition to annotating known compounds, molecular networking revealed related molecules that differed by oxidation, methylation, acetylation, hydroxylation, glycosylation, chain length, and saturation of alkyl chains, which enabled identification of previously undescribed metabolites of administered pharmaceuticals and microbial quinolones, as described below for azithromycin and Pseudomonas aeruginosa quinolones. The frequency of detection of the antibiotics across patients’ samples is shown in Fig. 3b, with corresponding clusters from the full network displayed for each antibiotic. The nodes in the antibiotic cluster represent the metabolic transformations of the antibiotic. Thus, molecular networking provides a glimpse into metabolic processes. The resulting molecular network revealed that among three patients, remarkably, only about 27.6% of detected molecular features were shared, highlighting the diversity of chemistry present in diseased human lungs (Fig. 3c). All three patients in this study had different mutations in the CFTR gene (see Materials and Methods), and patients 2 and 3 were diagnosed with CF-related diabetes. Two of the three patients (patient 1 and patient 3) suffered from chronic infections by Pseudomonas aeruginosa. Thus, various factors may play a role leading to the observed chemical diversity, which may arise from microbial (e.g., virulence and quorum sensing metabolites such as quinolones), host (e.g., bile acids, amino acids, sugars, eukaryotic lipids, fatty acids, sterols, peptides, immune-related molecules), and xenobiotic molecules. The diversity of these metabolites in CF sputum has been previously characterized (32), and many of the same compounds were found in the lung tissue in this study.

10.1128/mSystems.00375-19.3 FIG S3 Procrustes plots generated using principal-component analysis of the Jaccard distances for untargeted metabolomics data and unweighted UniFrac distances for 16s rRNA sequencing data. (a and b) Emperor visualization plots of metabolomics and closed-reference-picked OTUs. (c and d) Emperor visualization plots of metabolomics and deblurred sOTUs. (e) Percentages of shared mass spectrometry features between left and right lungs of all patients. Percentages were calculated by comparing the features that overlap to the total number of features associated with each pair of lungs. Download FIG S3, TIF file, 2.3 MB. Copyright © 2019 Melnik et al. 2019 Melnik et al. This content is distributed under the terms of the Creative Commons Attribution 4.0 International license.

A Procrustes analysis of metabolomics data and 16S rRNA data with closed-reference OTU picking revealed a close association between the microbiome and metabolome in the lung samples (Mantel test *r* statistic = 0.2409, *P < *0.001, *n *=* *277) (Fig. S3a and b). This analysis suggests that the microbial composition of each sample is associated in large part with the corresponding chemical diversity. Additionally, Procrustes analysis performed on metabolomics and 16S rRNA with deblurred sub-OTUs (sOTUs) (33) resulted in the same trend (Fig. S3c and d) (Mantel test *r* statistic = 0.2488, *P < *0.001, *n *=* *263). A PCoA plot of the metabolome data with Jaccard distance metric showed that a vast range of chemical diversity exists not only between the patients (Fig. 1c) but also within a patient’s own lungs (Fig. S3e). This suggests that the chemical makeup of the patients with CF disease is highly personalized and that a single CF lung contains unique chemical microenvironments that provide different niches for microbial pathogens to live in. While metabolic diversity between patients in relation to disease state was previously described (30, 32), the mechanisms leading to such diversity within the lungs remain poorly understood.

Two of the additional benefits of an untargeted metabolomics analysis approach are the ability to track the medications that are taken by the patient, as medical records can oftentimes be incomplete and/or inaccurate due to lack of patient compliance, and the ability to identify metabolic transformations of the medications. For example, in the present study, in addition to the prescribed medications listed in the clinical records (different antibiotics, bronchodilators, two medications for digestive health, medications given during surgery, and over-the-counter medications that are used as cough suppressants), antihistamines and multiple over-the-counter medications have been detected (Table 1). Detailed knowledge of extant exogenous compounds in tissues of interest is important, among other reasons, for evaluation of their effect on the microbiome and microbial interactions for better understanding disease etiology. Another advantage of a molecular networking approach for untargeted metabolomics data analysis is that it allows postulating structures for unknown compounds, nodes of which are connected to nodes of known compounds (annotation propagation), and is therefore very useful for identifying drug metabolites (Fig. 3b). The distributions of drugs and the metabolites can then be evaluated by 3D cartography even in the absence of a stable isotope tracer. By the use of a molecular networking approach in this study, unknown metabolites that had never before been reported in blood or tissue of humans and animals were detected (Fig. S4). The unknown metabolite of azithromycin (*m/z* 382.26) is annotated as methylated-azithromycin, where the methylation, based on the analysis of the fragmentation data, occurs in the core macrolide ring of azithromycin and another unknown metabolite is proposed to have oxidation in the macrolide ring (Fig. S4). These modifications of the core macrolide structure of azithromycin have not been described previously, and their biological activities are unknown. Although these metabolites were not detected in *in vitro* cultures of microbes isolated from these patients in the presence of azithromycin, the possibility that these are microbially derived warrants further investigation and cannot be ruled out. Specific *in vivo* conditions may be necessary for regulation of microbial genes involved in antimicrobial metabolism.

**TABLE 1:**
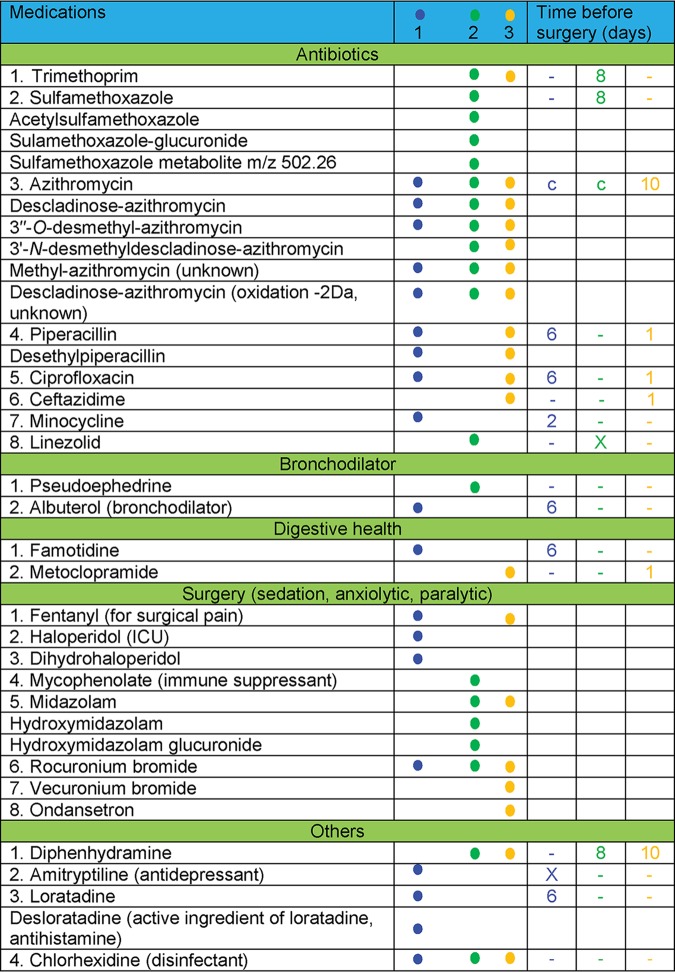
Medications detected in the MS data and time of administration prior to the day of lung explantation surgery^*a*^

a c, continuously administered. The numbers 1, 2, and 3 at the top of column 2 and the data in columns 3, 4, and 5 represent patient 1, patient 2, and patient 3, respectively. Dashes indicate that the drug was not prescribed.

10.1128/mSystems.00375-19.4 FIG S4 Results of mass spectral analysis of azithromycin and its proposed metabolites are shown. Download FIG S4, TIF file, 1.4 MB. Copyright © 2019 Melnik et al. 2019 Melnik et al. This content is distributed under the terms of the Creative Commons Attribution 4.0 International license.

**FIG 4:**
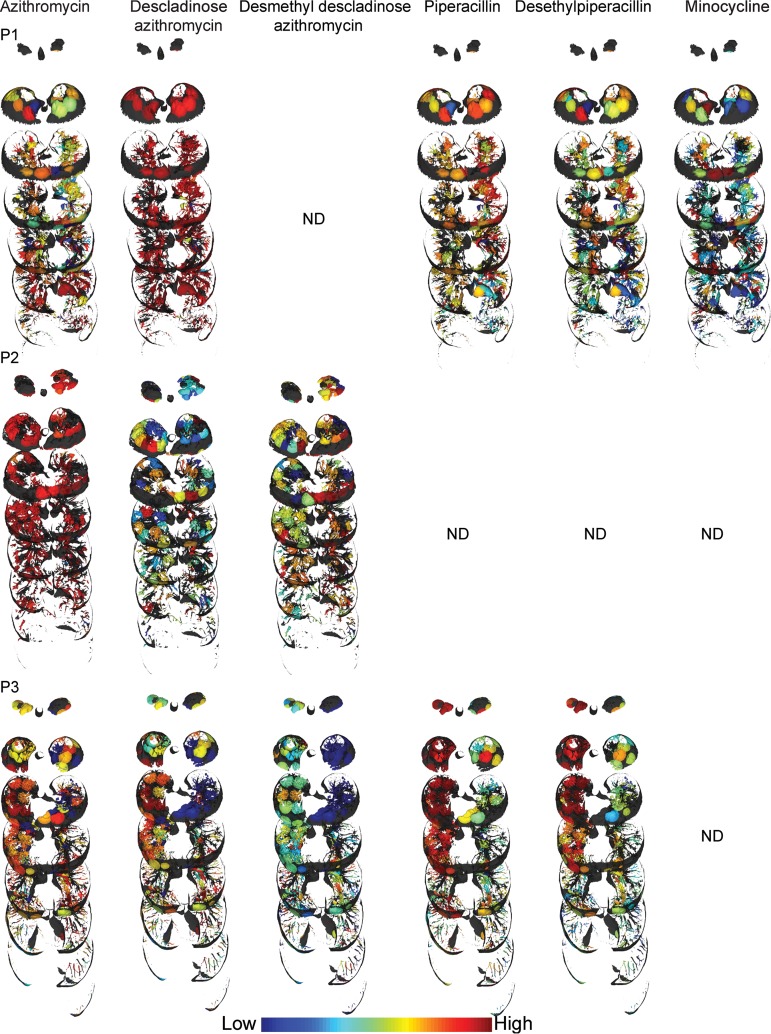
Distribution of selected antibiotics and the metabolites. P1, patient 1; P2, patient 2; P3, patient 3; ND, not detected. An intensity scale is provided at the bottom (distributions of additional antibiotics and their metabolites are shown in Fig. S5). The relative distributions should be compared within a patient lung. Full visualizations of metabolite maps can be accessed via the following hyperlinks: patient 1, patient 2, and patient 3.

As with the microbial heterogeneity, we have observed differences in metabolome distributions. Molecular networking and 3D volume cartography of the antibiotics revealed patient-specific metabolism and drug distributions (Fig. 4; see also Fig. S5). The distributions of antibiotics were also found to be different between the left and right lungs of the same patient. For example, the antibiotic piperacillin and its metabolites were abundant in the upper lobes of the right lung of patient 3 but present in relatively lower abundance in the left lung of this patient (Fig. 4). In patient 3, there was higher penetration of piperacillin in the upper and middle lobes and poor penetration in the lower lobes of both lungs. Similarly, the antibiotic linezolid detected in patient 2 had lower relative abundance in the lower lobe of the right lung (Fig. S5). Overall, the drug metabolites largely follow the same distribution as the parent drug except for the glucuronidated metabolite of sulfamethoxazole (Fig. S5), indicating that metabolism may not be a significant contributing factor for the observed uneven distribution of detected antibiotics. Differential levels of vascularization and tissue necrosis also contribute to nonuniform drug penetration in severe end-stage CF disease.

10.1128/mSystems.00375-19.5 FIG S5 Distribution of antibiotics and its metabolites in patients. (a) Patient 1. (b) Patient 2. (c) Patient 3. Download FIG S5, TIF file, 2.3 MB. Copyright © 2019 Melnik et al. 2019 Melnik et al. This content is distributed under the terms of the Creative Commons Attribution 4.0 International license.

**FIG 5:**
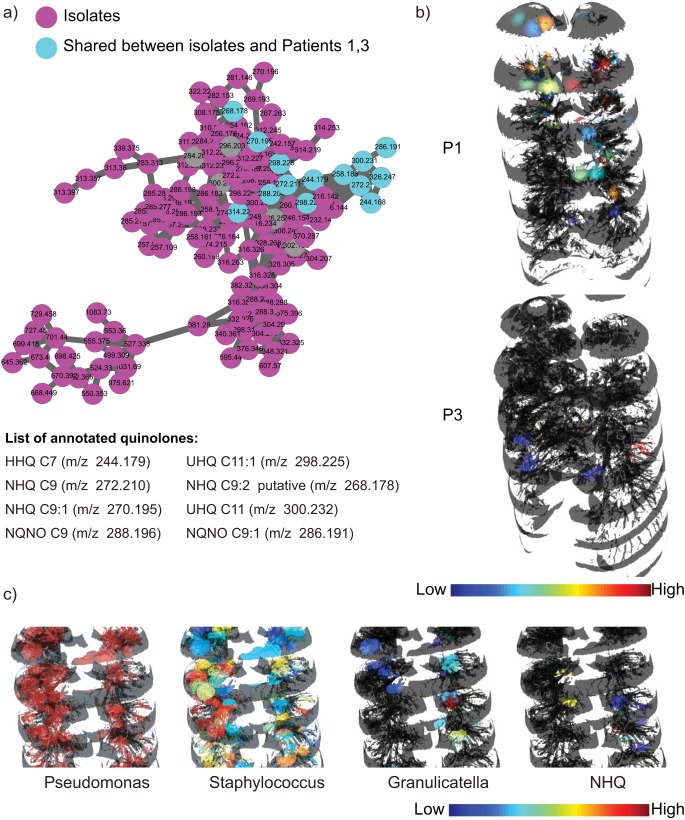
Molecules produced by P. aeruginosa in patients 1 and 3. (a) The molecular network cluster of quinolones detected in the lung tissue of patients 1 and 3 and *in vitro* microbial cultures of *Pseudomonas* isolated from sputum and the swabs collected from lung sections is shown. (b) The distributions of the quinolone HHQ are shown for patients 1 and 3. All the other quinolones showed similar distributions in those patients (Fig. S8). (c) Inset views of the distribution of *Pseudomonas*, *Staphylococcus*, *Granulicatella*, and the *Pseudomonas* quinolone NHQ in patient 3 suggestive of upregulation in quinolone production by *Pseudomonas* in the regions where interactions of *Pseudomonas* with *Staphylococcus* and *Granulicatella* and possibly other microbes take place. In agreement with this observation, the levels of production of HHQ and NHQ were also found to increase in cocultures of *Pseudomonas* and *Staphylococcus* compared to *Pseudomonas* grown alone under identical conditions (Fig. S9).

To directly link microbiome and metabolome information and identify associations of compounds detected in the lung tissue with specific microbes, isolates from lungs of all patients were obtained from the clinical laboratory and cultured directly from swabs of the lung tissue and MS data were acquired on the organic extracts of the *in vitro* cultures by the use as the same protocol as that employed for the tissue extracts. Molecular networking of MS/MS data from culture extracts and tissue extracts provided insights into the molecules that are shared between microbes and the human host (Fig. S6). These molecules included microbe-specific virulence factors, as well as various other molecules such as lipids, fatty acids, amino acid metabolites, dipeptides, and tripeptides. Similarly to our previously reported observation for one CF lung of a single CF patient (16, 32), a larger diversity of quinolones was detected in cultured isolates than in the lung tissue of all patients whose lungs were dominated by *Pseudomonas* in this study, including a quinolone at *m/z* 268.170 that was never reported before (Fig. 5). On the basis of MS (MS^1^) and tandem MS (MS^2^) data, the structure of this quinolone is proposed to contain two double bonds in the alkyl side chain as opposed to the single double bond found in unsaturated quinolones described in the literature (34) (Fig. S7). Similarly to previous reports on patient sputum (32) and lung tissue (16), the *Pseudomonas* quinolone signal (PQS) was not detected in the lungs of patients 1 and 3. To gain further insight into the variation in the distribution of quinolones in the patients whose lungs were dominated by *Pseudomonas*, we investigated the distribution of quinolones directly within the lungs of these patients (Fig. 5b; see also Fig. S8). Previously, we reported that the quinolones were prevalent at the upper lobe of the left lung of a single patient (16). In the present study, quinolones were found to be exclusively present at the upper lobe of lungs of patient 1 and only in the middle of the lungs of patient 3. This indicates that the patients whose lungs were dominated by *Pseudomonas* showed individualized phenotypes with respect to the expression of these quorum sensing molecules. Furthermore, rhamnolipids, the *Pseudomonas* biosurfactant, were not detected in the lungs of patients 1 and 3 in this study but were detected in our previous study (16). Patient-specific production of rhamnolipids has been reported previously by culturing isolates in the laboratory but not directly from infected tissue (13). Such compartmentalization of microbial activity within patients, as well as variations between patients, is a hallmark of complexity that is inherent to polymicrobial infection in a complex organ, such as, in the present case, a CF lung. Direct visualization of the individual phenotypes in diseased organs enables informed understanding of divergent evolution as well as of the spatial molecular environment within a host.

10.1128/mSystems.00375-19.6 FIG S6 Molecular network analysis of all six lungs from the three patients and of microbial isolates. The molecular network is color coded by sample source. Download FIG S6, TIF file, 2 MB. Copyright © 2019 Melnik et al. 2019 Melnik et al. This content is distributed under the terms of the Creative Commons Attribution 4.0 International license.

10.1128/mSystems.00375-19.7 FIG S7 (a and b) Extracted ion chromatograms (EIC) (a) and mass spectra and structures (b) of quinolones NHQ, HHQ, NHQ-C9:1db, and NHQ-C9:2db (proposed) are shown. The position of additional double bond for NHQ-C9:2db is not known and is putatively drawn. (c) Structures of fragments described previously in the literature for NHQ, HHQ, and NHQ-C9:1db. Proposed structures for fragments corresponding to NHQ-C9:2db are enclosed in brackets. Download FIG S7, TIF file, 2.7 MB. Copyright © 2019 Melnik et al. 2019 Melnik et al. This content is distributed under the terms of the Creative Commons Attribution 4.0 International license.

10.1128/mSystems.00375-19.8 FIG S8 Distribution of quinolones that are common to patients 1 and 3. Download FIG S8, TIF file, 2.3 MB. Copyright © 2019 Melnik et al. 2019 Melnik et al. This content is distributed under the terms of the Creative Commons Attribution 4.0 International license.

10.1128/mSystems.00375-19.9 FIG S9 Bar graphs of plots of areas under the curve normalized to total ion current (error bars indicate standard deviations from mean values representing results from three independent experiments [*n *=* *3]). (a) Levels of quinolone production by Pseudomonas aeruginosa in single-species culture and in mixed culture with Staphylococcus aureus are shown. PS1 represents cultures that were mixed in a ratio of 1:1; PS10 represents a ratio of 1:10, where S. aureus culture was diluted 10-fold. (b) Levels of production of quinolone NHQ in response to antibiotic exposure by Pseudomonas aeruginosa isolated from patient 3. Each color represents a different concentration of antibiotics. Download FIG S9, TIF file, 1.9 MB. Copyright © 2019 Melnik et al. 2019 Melnik et al. This content is distributed under the terms of the Creative Commons Attribution 4.0 International license.

The spatial codistributions of microorganisms, antibiotics, and microbial molecules were investigated to establish microbe-metabolite interactions. Although the presence of a single dominant pathogen renders correlation analysis rather uninformative, several trends have been observed. In particular, the distributions of certain microorganisms such as *Staphylococcus* and *Granulicatella* were found to be associated with the distribution of quinolones produced by *Pseudomonas* in patient 3, as shown for 2-nonyl-4(1H)-quinolone (NHQ) in Fig. 5c. We have recently shown that the presence of Staphylococcus aureus isolated from a CF patient resulted in increased quinolone and biofilm production by coisolated *Pseudomonas in vitro* (35). Similarly, mixing cultures of *Pseudomonas* and *Staphylococcus* isolated from patient 3 in this study resulted in increased production of 4-hydroxy-2-heptylquinoline (HHQ) and NHQ compared to the levels seen with *Pseudomonas* grown alone under identical conditions (Fig. S9a). This observation indicates that the production of quinolone molecules is also modulated in part by the microbial interactions present in a polymicrobial infection. The complexity of these microbial interactions is further increased as antibiotics cause perturbations of microbial communities reflected by suppression of the virulence factors. Variation in production of quinolones by patient isolates of *Pseudomonas* was observed upon exposure to sub-MICs (Fig. S9b). This, together with the other observations reported here, supports the hypothesis that genetic changes may not be the only factors responsible for changing metabolism and that microbial interactions, in conjunction with multiple other factors, including sub-MICs of antibiotics and perhaps other xenobiotics, may also play a role, thus calling for the design of specific studies investigating these phenomena in multiple patient isolates. Thus, it is reasonable to hypothesize that both specific microbial interactions in the lungs and differential abundances of antibiotics could result in metabolic divergences, creating isolated regions of enhanced biofilm formation and tissue damage in CF patients such as are often revealed by chest X-rays and CT scans. Application of advanced techniques such as ultra-high-resolution computed tomography in conjunction with the approach presented here could be a focus of future studies (36).

### Conclusion.

Cystic fibrosis is a devastating genetic disease affecting tens of thousands of people worldwide. In this work, we have presented findings of spatial distributions of microbes, medications, and their metabolites throughout lungs of three patients afflicted with CF. We have found that although the microbiome is predominantly patient specific, the chemical differences between locations within patient’s own lungs may be greater than interpatient variations. In-depth analyses revealed differential drug penetration, metabolism of prescribed medications, and microbial compartmentalization resulting in metabolic divergence governed by local microbial interactions. Mapping of microbial communities and localized chemistries allowed visualization of interactions among community members, such as production of quinolones by *Pseudomonas* when present in a community structure with other microbes such as *Staphylococcus* or *Granulicatella.* Visualization of such local infection loci highlights the importance of development of effective drug delivery approaches. Considering recent advances in the development of small-scale robots (as small as a few micrometers in size) that can noninvasively access confined spaces (37), targeted access of internal tissues as well as precision delivery of drug payloads may become feasible in the near future. In general, a paradigm shift of considering localized regions of divergent microbial and chemical distributions is an important next step for effective disease management of polymicrobial infections.

## MATERIALS AND METHODS

### Tissue collection and processing.

To map the microbiome and metabolome of explanted lungs in 3D, the lungs of three patients were obtained in close coordination with the patient’s physician and the surgical team. This work was approved by the University of California (UC) Institutional Review Board (project no. 081500), and informed consents were obtained prior to tissue collection. The CFTR mutation in patient 1 was dF508/G551D (with no clinical diabetes), in patient 2 was dF508/3120 + 1G>A (with observed clinical diabetes), and in patient 3 was dF508/dF508 (with observed clinical diabetes). The general workflow for tissue sectioning was described previously (16). Briefly, both the right and left lungs were collected from subjects 1, 2, and 3. The tissue sectioning was performed at the hospital under the guidance of a pathologist. The lungs were first sliced horizontally. The anatomical orientation of each slice was recorded. Every alternate slice starting from the apex of the lung was further subsectioned into small sections 1 to 2 cm^3^ in size, maintaining the recorded orientation. Each of the subsectioned tissue pieces was swabbed with sterile soft foam swabs moistened with Tris-EDTA (pH 7.4). The swabs were stored in 96-well bead plates from a PowerSoil-htp 96-well soil DNA isolation kit. The plate was placed on dry ice prior to and during the collection step. The individual tissue pieces were stored in glass jars placed on dry ice. The samples were kept frozen at −80°C until further processing. Bacterial DNA was isolated from the swabs using a PowerSoil-htp 96-well soil DNA isolation kit following the manufacturer’s instructions and was subjected to prokaryotic ribosomal 16S rRNA-based sequencing using the standardized Earth Microbiome Protocol (http://www.earthmicrobiome.org/protocols-and-standards/). Amplicons were cleaned, pooled, and then sequenced on an Illumina MiSeq sequencer. Because the lungs were obtained at different times, the sequences analyzed for this study were obtained from two individual sequencing runs (sequencing run 1, patients 1 and 2; sequencing run 2, patient 3). The sequencing runs were performed at the Genomics Center of the Institute for Genomic Medicine of the University of California, San Diego (UC San Diego). For untargeted metabolomics analysis, the tissue sections were weighed and extracted with 1 ml/g of tissue with a 2:2:1 mixture of ethyl acetate, methanol, and water. An aliquot of 150 μl of the extract was dried for each tissue section and analyzed by MS.

### MS data acquisition.

The tissue extracts and extracts of bacterial isolates from the subjects were cultured on sheep blood agar and MacConkey agar and were then resuspended in 80% methanol containing 1 μM sulfadimethoxine and analyzed with a UltiMate 3000 ultra-high-performance liquid chromatography (UHPLC) system (Thermo Scientific) using a Kinetex C_18_ reversed-phase UHPLC column (50 by 2.1 mm, 1.7-μm pore size) and Maxis quadrupole-time of flight (Q-TOF) mass spectrometer (Bruker Daltonics) equipped with an electrospray ionization (ESI) source. The column was equilibrated with 2% solvent B (98% acetonitrile, 0.1% formic acid, and LC-MS-grade water, with solvent A as 0.1% formic acid in water) for 1 min followed by a linear gradient from 2% solvent B to 100% solvent B over 10 min and then by a hold at 100% solvent B for 2.5 min. A small wash segment was employed to wash the column (100% solvent B for 0.5 min, 100% to 10% solvent B over 0.5 min), following which the column was kept at 2% solvent B for 1 min at a flow rate of 0.5 ml/min throughout the run. MS spectra were acquired in positive-ion mode in the range of 50 to 2,000 *m/z*. A mixture of sulfamethazine, sulfamethizole, sulfachloropyridazine, sulfadimethoxine, amitriptyline, and coumarin-314 (10 μg/ml each) was run after every eight injections for quality control. An external calibration was performed with ESI-L low-concentration tuning mix (Agilent Technologies) prior to data collection, and Hexakis(1H,1H,3H-tertrafluoropropoxy)phosphazene was used as an internal calibrant throughout the runs. A capillary voltage of 4,500 V, a nebulizer gas (nitrogen) pressure of 2 bar, an ion source temperature of 200°C, a dry gas flow of 9 liters/min at source temperature, and spectral rates of 3 Hz for MS^1^ and 10 Hz for MS^2^ were used. For acquiring MS/MS fragmentation patterns, the 10 ions showing the highest level of signal intensity per MS^1^ were selected and fragmented. A basic stepping of collision radio frequency (RF) values of 550 and 800 peak-to-peak voltage (Vpp) with a timing of 50% for each step and transfer time stepping of 57 and 90 μs with a timing of 50% for each step was employed. The MS/MS active exclusion parameter value was set to 3, with release after 30 s. The mass of the internal calibrant was excluded from the MS/MS list, and a mass range of *m/z* 921.5 to 924.5 was used.

The microbial isolates of Pseudomonas aeruginosa, Staphylococcus aureus, and Stenotrophomonas maltophilia collected from the patients were obtained from the Center of Advanced Clinical Medicine, UC San Diego. The culturing of the isolates and the extractions were performed as described previously (16). The MS data were collected using the same conditions as described above for lung tissue.

### LC-MS/MS data analysis.

All mzXML files were cropped with an *m/z* range of 50.00 to 2,000.00 Da and a retention time (RT) range of 0.5 to 18.5 min. Feature extraction was performed using MZmine2 (http://mzmine.sourceforge.net/) with a signal height threshold of 5.0e3 (38). The mass tolerance was set to 10 ppm, and the maximum allowed retention time deviation was set to 0.01 min. For chromatographic deconvolution, the local minimum search algorithm was used with a minimum relative peak height of 1% and a minimum retention time range of 0.01 min. The maximum peak width was set to 1 min. After isotope peak removal, the peaks in the lists of all samples were aligned with the retention time and mass tolerances mentioned above. After the creation of a feature matrix containing the feature retention times and the exact masses and peak areas of the corresponding extracted ion chromatograms, the metadata of the samples were added. The signal intensities of the features were normalized (using probabilistic quotient normalization [PQN]) (39).

Statistical analysis was carried out as follows. QIIME 2 was used to perform principal-coordinate analysis (PCoA) (Jaccard distance metric). The PCoA plots were visualized in EMPeror (40).

### Molecular networking.

**(i) Explanation of molecular networking analysis.** In a mass spectrometer, the MS/MS spectra acquired with identical parameters of collision energy are highly similar, if not identical. For the majority of compounds, the spectra do not significantly differ even with small variations in collision energy. Thus, automated matching of experimental MS/MS spectra with MS/MS spectra available in spectral libraries is routinely performed to annotate known molecules. Molecular networking algorithms further extend the capability of spectral comparisons, and their use is based on the fact that molecules that are similar in structure and contain common substructural motifs fragment similarly in a mass spectrometer. Thus, molecules that differ in the presence of small functional groups such as additional methyl groups, hydroxyl groups, and sugar groups and with respect to saturation of chemical bonds, cyclization, etc., have similar MS/MS spectra. This similarity in MS/MS spectra is quantified by spectral alignment, and the result is assigned a similarity score. The output is displayed as nodes connected by edges. Here, the nodes that are connected to each other represent molecules that are structural analogs. The nodes that are not connected to each other represent molecules that deviate significantly with respect to their structural similarity. Simply put, a cluster of connected nodes represents a structurally related molecular family. For example, the drug sulfamethoxazole and its glucuroniated counterpart consist of structurally similar molecules with similar fragmentation patterns and hence are displayed as two connected nodes. Furthermore, all identical MS/MS spectra from different samples are combined under one node. This allows rapid and efficient comparisons of data to identify molecules that are common between samples and molecules that are unique. In summary, molecular networking allows one to annotate known molecules, to predict analogs of known molecules, and to annotate biotransformations of known molecules and allows comparisons across samples and across data sets. Detailed descriptions of molecular networking fundamentals and use are available at https://ccms-ucsd.github.io/GNPSDocumentation/massspecbackground/networkingtheory/.

**(ii) Parameters.** The molecular network was created using the online workflow at the GNPS platform. The data were then clustered with MS-Cluster with a parent mass tolerance of 0.1 Da and a MS/MS fragment ion tolerance of 0.1 Da to create consensus spectra. Further, consensus spectra that contained less than 3 spectra were discarded. A network was then created where edges were filtered to have a cosine score above 0.7 and more than 4 matched peaks. The edges between two nodes were kept in the network if and only if each of the nodes appeared in the list of respective top 10 most similar nodes of the other. The spectra in the network were then searched against GNPS’s spectral libraries. All matches kept between network spectra and library spectra were required to have a score above 0.7 and at least 4 matched peaks. The molecular networks and the parameters used are available at the hyperlinks below (see “Data availability”).

In total, 1,776 (7.8%) of the nodes were annotated, representing a rate higher than the typical rate of annotations of 1.8% in an untargeted metabolomics experiment (30). This difference is likely a consequence of the fact that many of the reference MS/MS libraries in the public domain were populated from studies of human samples and contain most of the therapeutics used in the clinic. The error rates of these annotations have been assessed by the GNPS community; with the scoring settings used to obtain the annotations, 1% were classified as incorrect, 4% were classified as having insufficient information available, and 4% were classified as representing an isomer or correct, while 91% were presumed to be correct (30).

### 16S rRNA gene analysis.

As described above, sequences were obtained over the course of 2 months through two independent sequencing runs. The samples for patient 1 and patient 2 were sequenced in one batch, and samples for patient 3 were sequenced separately. All sets of sequences were processed and analyzed using Qiita (41). First, the sequencing runs were quality trimmed and filtered using default parameters, resulting in 15,629,914 sequences with a mean length of 150 nucleotides. Next, after the sequences were trimmed (at 150 nucleotides), they clustered into operational taxonomic units (OTUs) using the closed reference OTU picking method at 97% sequence similarity. UCLUST was used as the underlying clustering algorithm, and Greengenes (August 2013 release) was the reference database used (42). This resulted in 340 samples with a mean of 25,078 sequences per sample. After rarefaction at 3,369 sequences per sample, 277 samples were used for downstream analyses, including the creation of taxonomy summaries and the calculation of the unweighted and weighted UniFrac distances. The most abundant OTU for patient 2 was identified as representing an unclassified genus in the family *Xanthomonadaceae*. BLAST analysis of the sequence corresponding to this OTU from patient 2 revealed that it belongs to the genus *Stenotrophomonas*. As controls, a total of 49 wells (either containing a blank swab or empty) were interleaved between each of the two sampling sites (left lung and right lung) of the three subjects. The vast majority of the samples (72%) yielded zero sequences. The remaining 14 samples had a nonzero amount of sequences. Of these, 7 samples were represented by fewer than 4 sequences, a negligible amount compared to the 3,500 sequences per sample used for analysis. And the last 7 samples were represented by over 6,000 sequences each. Although the last sample set was processed without any DNA, the well-to-well contamination that occurred during the DNA extraction step yielded these sequences. We removed these samples since the DNA was biological and not representative of a type of actionable contamination (43, 44).

For statistical analysis, QIIME2 (45) was used to compute the PCoA data and the weighted and unweighted UniFrac distances (weighted UniFrac distances were described previously [46]) and Procrustes analysis with metabolomics data. The PCoA and Procrustes plots were visualized in EMPeror (40). The Mantel test was used to calculate *r*^2^ scores from comparisons between mass spectrometry data and for both closed-reference and deblur 16S rRNA gene analysis data using scikit-bio’s 0.5.5 Mantel’s test implementation.

### 3D lung model generation and visualization.

The procedure for creation and visualization of 3D models has been previously described (16). Briefly, the CT-scan images obtained from the radiology department at the Hillcrest hospital in San Diego were combined to create a 3D lung model and the data were exported in the .stl format using InVesalius 3.0. The extraneous pixels corresponding to the chest and back of each model were manually deleted using Geomagic Wrap 3D modeling software. The relative abundances of detected microbes and molecules were plotted onto these models using a modified version of the `ili software available at http://mwang87.github.io/ili/ (16, 47).

### Data availability.

All data presented in this article are publicly available. The molecular network analysis and parameters for the patient data are available at https://gnps.ucsd.edu/ProteoSAFe/status.jsp?task=6f92a21af31d4569bcdb3cce803c600c. The molecular network analysis and parameters for the patient data and data acquired on cultured microbial isolates are available at https://gnps.ucsd.edu/ProteoSAFe/status.jsp?task=45d70e56faae4081bbba1f7a9ce38019. All raw and processed 16S amplicon sequencing data and metadata are available with Qiita study identification no. 10169 and as an EBI study with accession no. ERP110498. All figures in this article have associated raw data available through the accession numbers given above. The code for 3D mapping via the browser tool github `ili is available at https://github.com/mwang87/ili, and the tool can be accessed via the hyperlink http://mwang87.github.io/ili/. The data determined in the analyses of MS/MS fragmentation patterns were deposited in an online repository, namely, MassIVE (https://massive.ucsd.edu/ProteoSAFe/static/massive.jsp), and are available under identifiers (ID) MSV000079652 and MSV000079398.
