# Supplementary figures and images for: Molecular and Microbial Microenvironments in Chronically Diseased Lungs Associated with Cystic Fibrosis
Source: mSystems. 2019 Sep 24;4(5):e00375-19. doi: 10.1128/mSystems.00375-19 (PMC6759567; doi:10.1128/mSystems.00375-19)

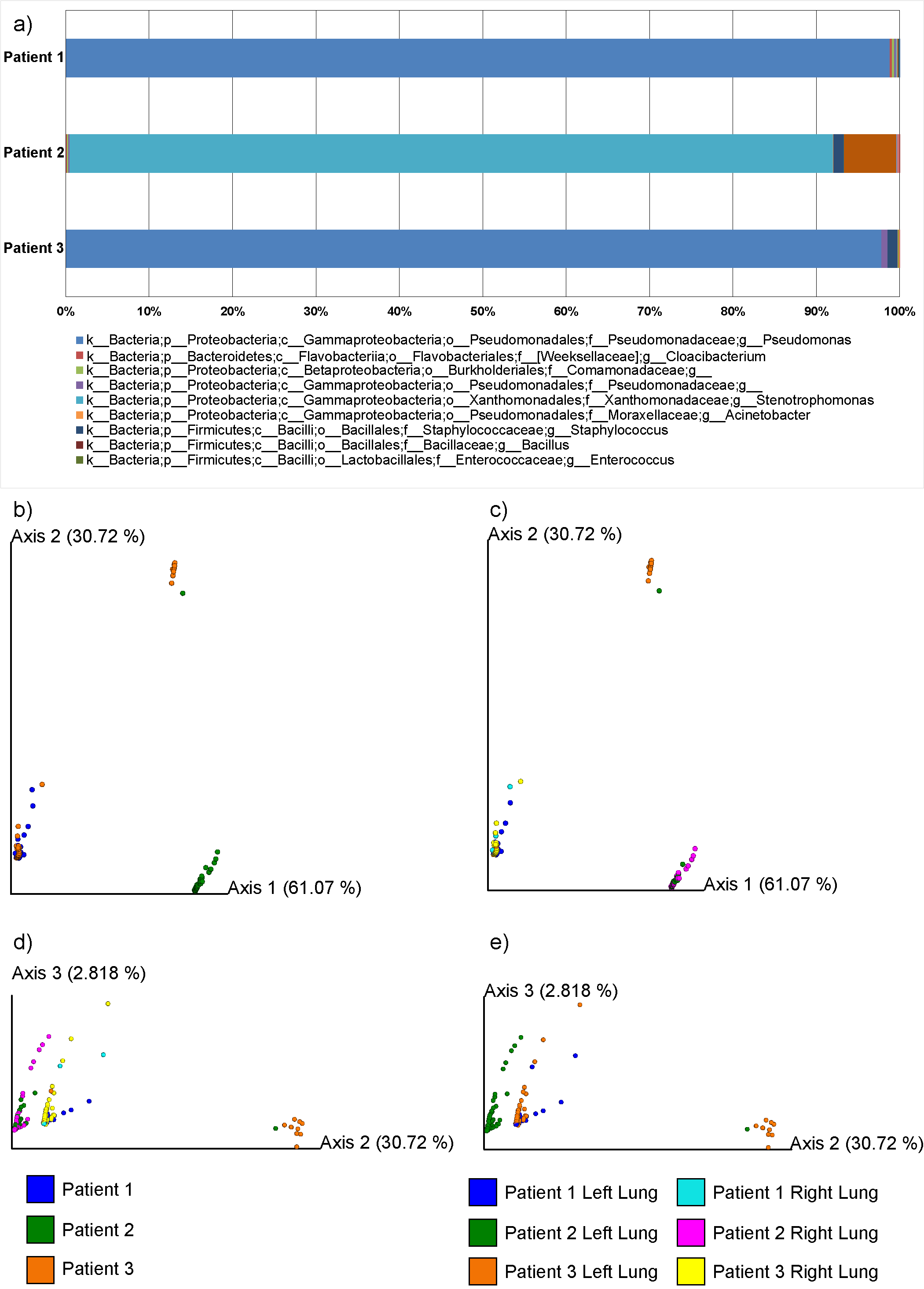

Supplement: FIG S1 [file mSystems.00375-19-sf001.tif]

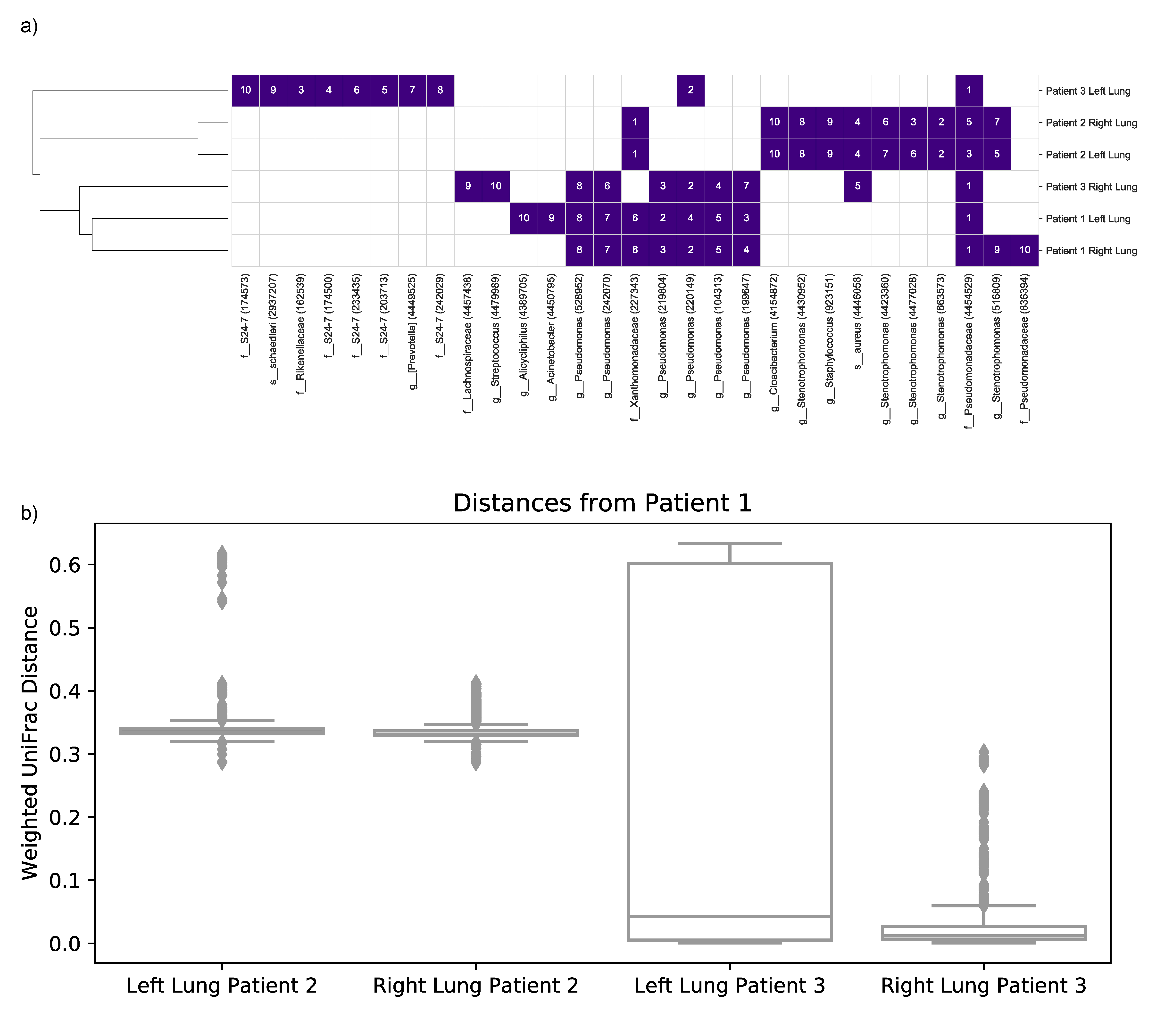

Supplement: FIG S2 [file mSystems.00375-19-sf002.tif]

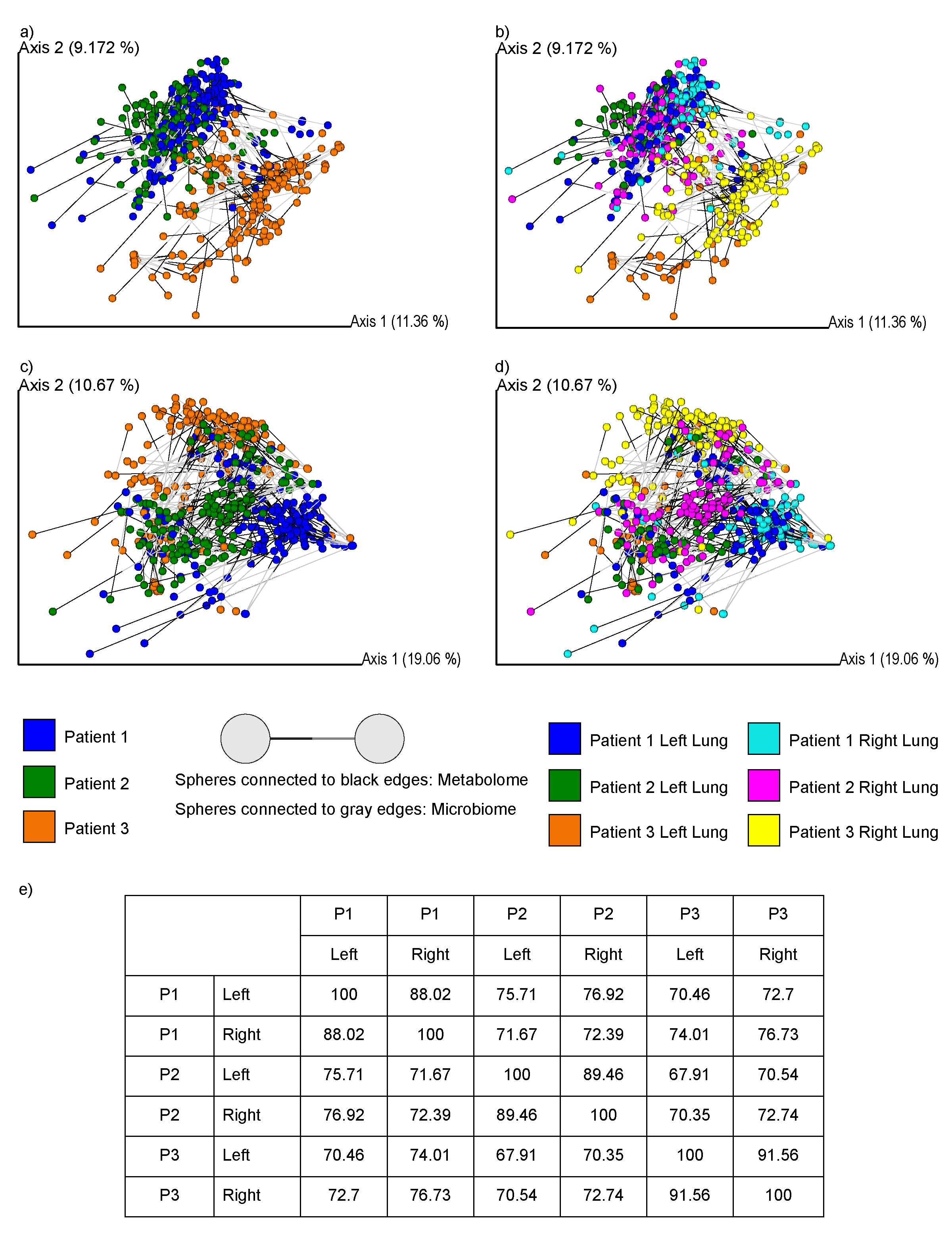

Supplement: FIG S3 [file mSystems.00375-19-sf003.tif]

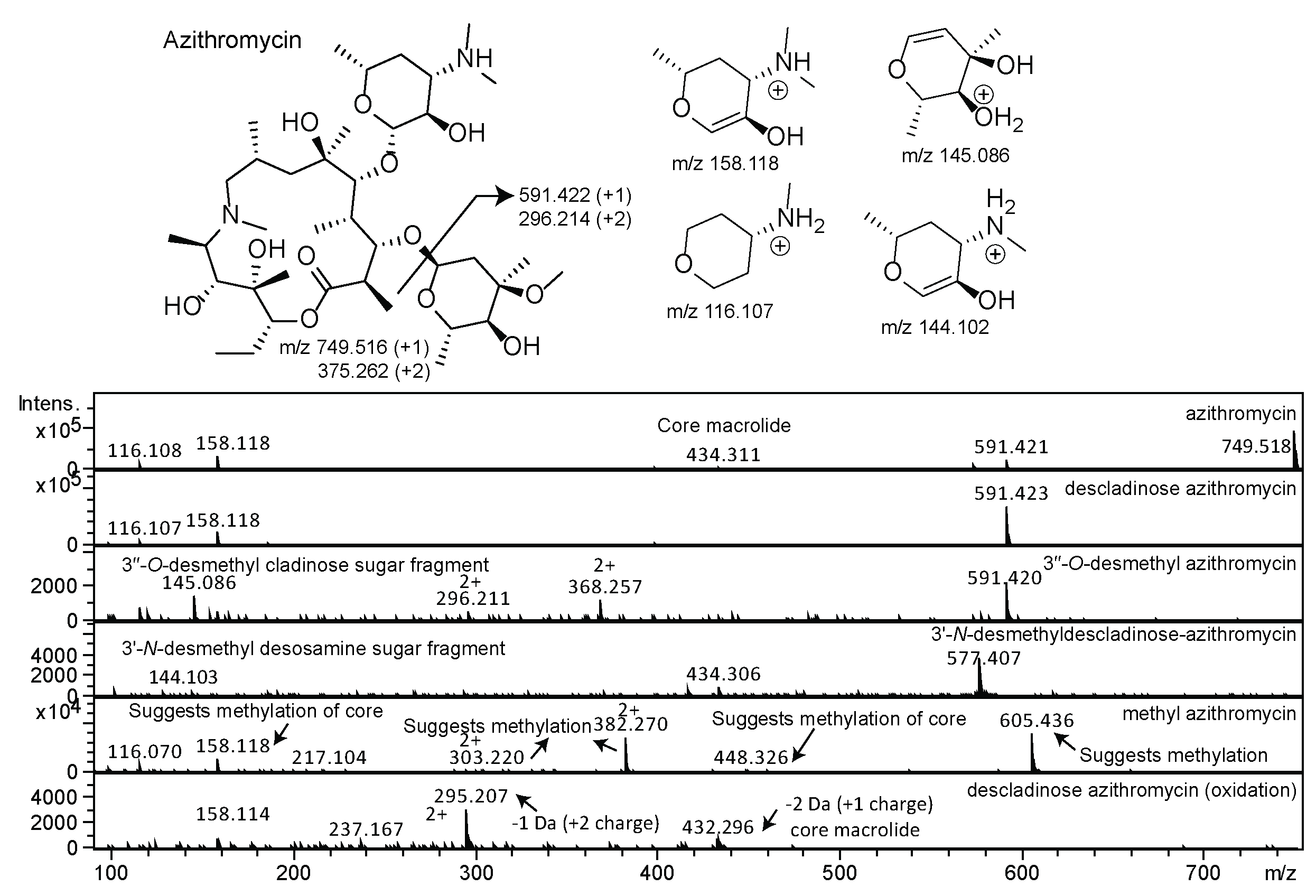

Supplement: FIG S4 [file mSystems.00375-19-sf004.tif]

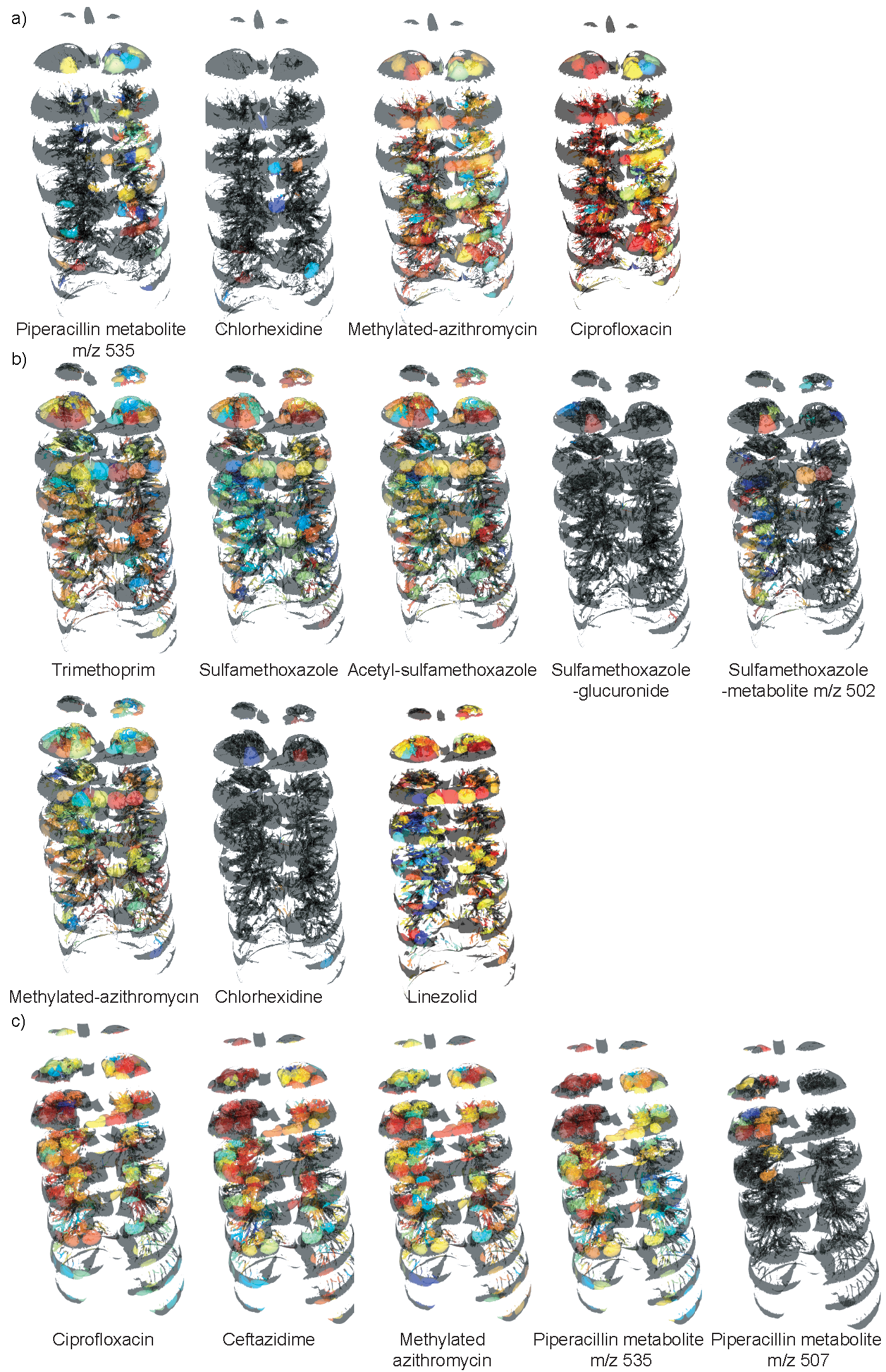

Supplement: FIG S5 [file mSystems.00375-19-sf005.tif]

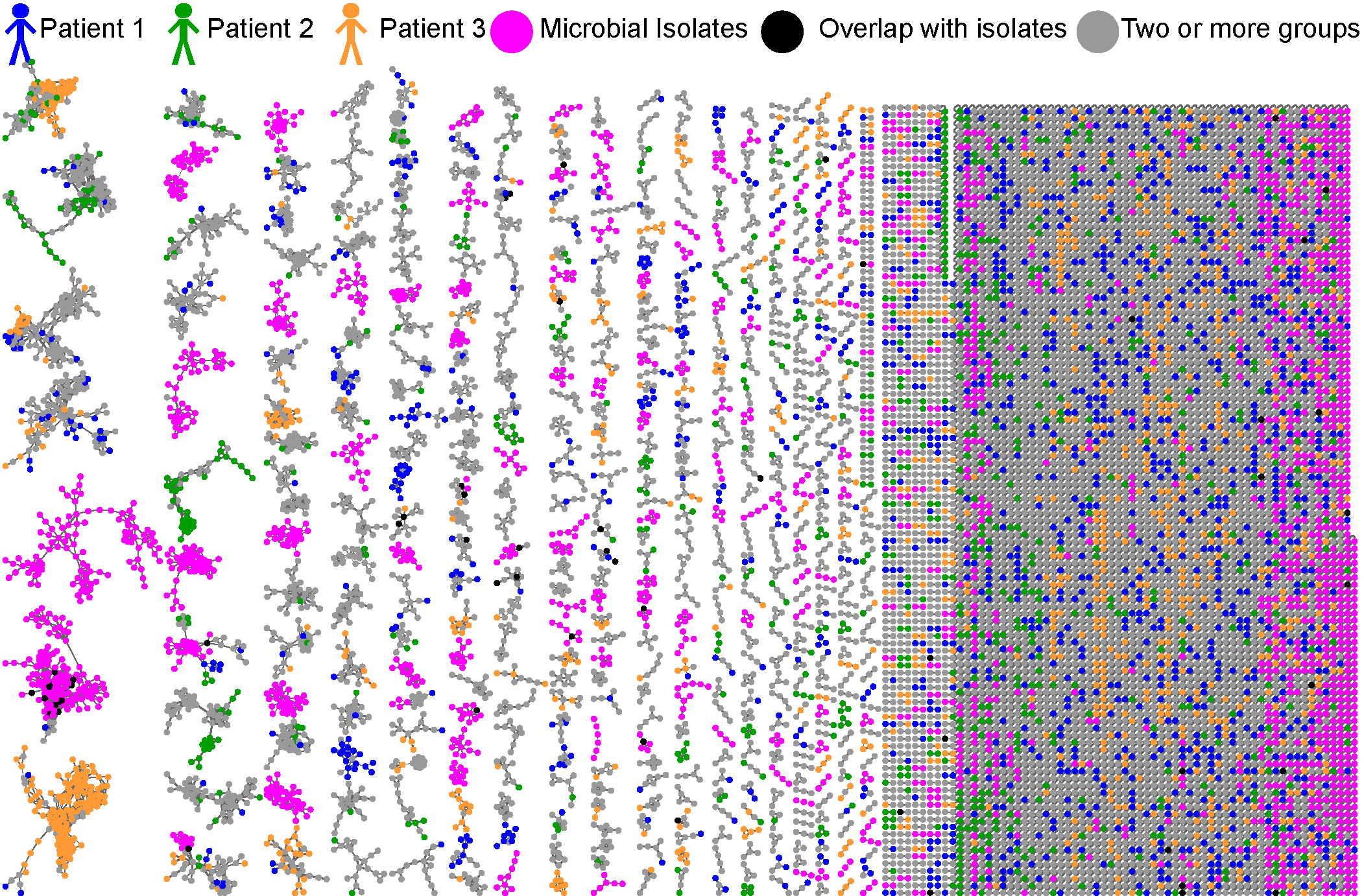

Supplement: FIG S6 [file mSystems.00375-19-sf006.tif]

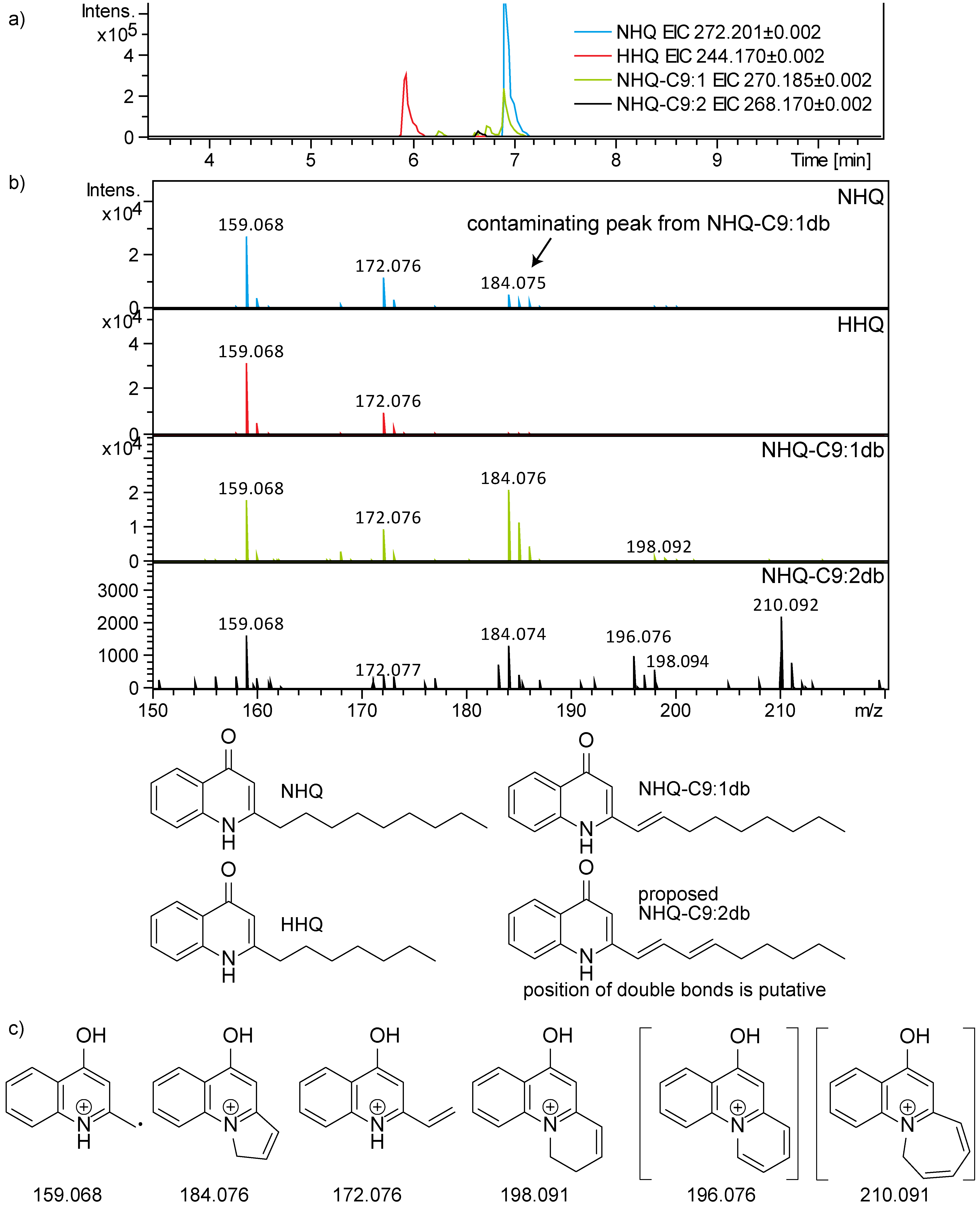

Supplement: FIG S7 [file mSystems.00375-19-sf007.tif]

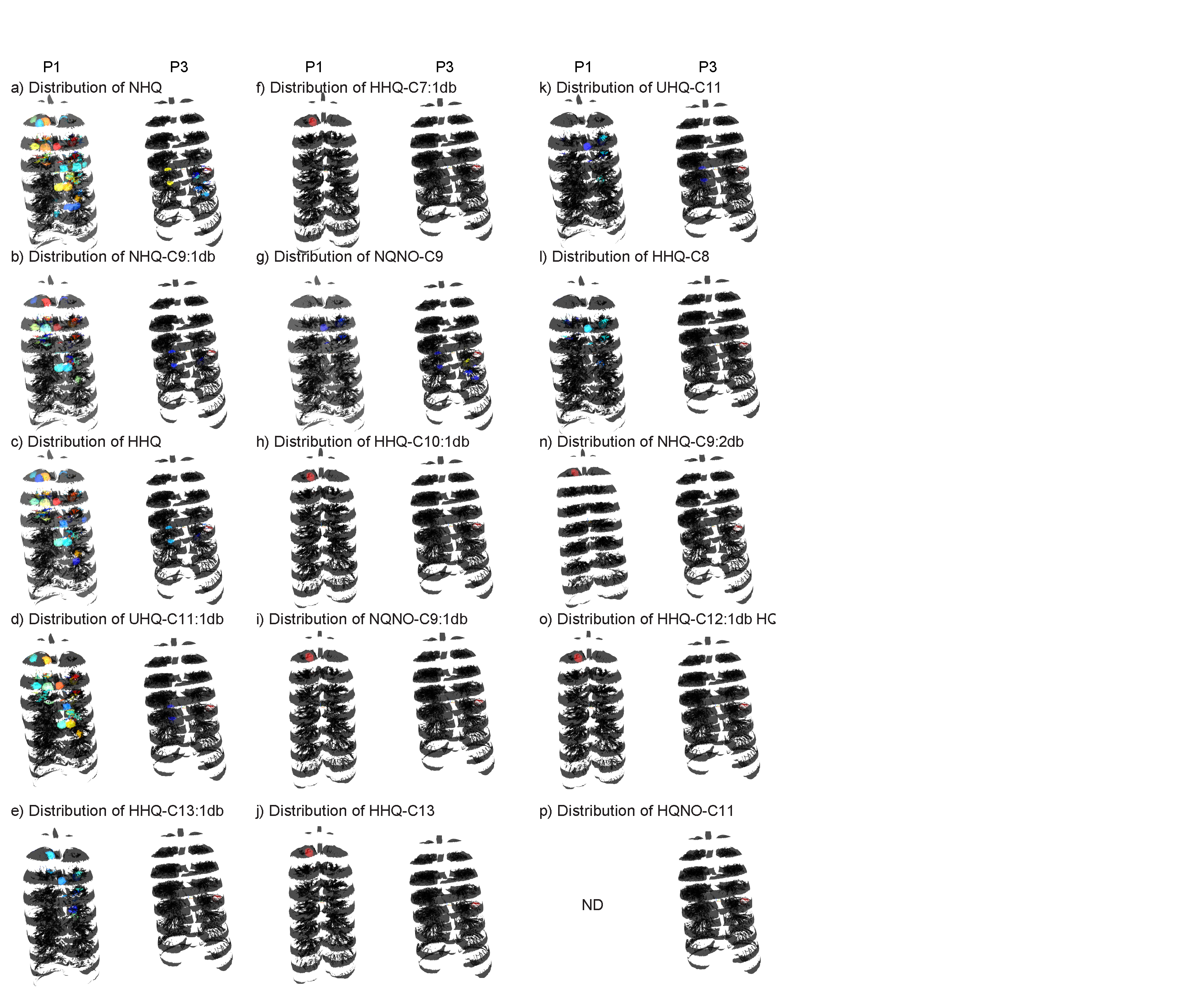

Supplement: FIG S8 [file mSystems.00375-19-sf008.tif]

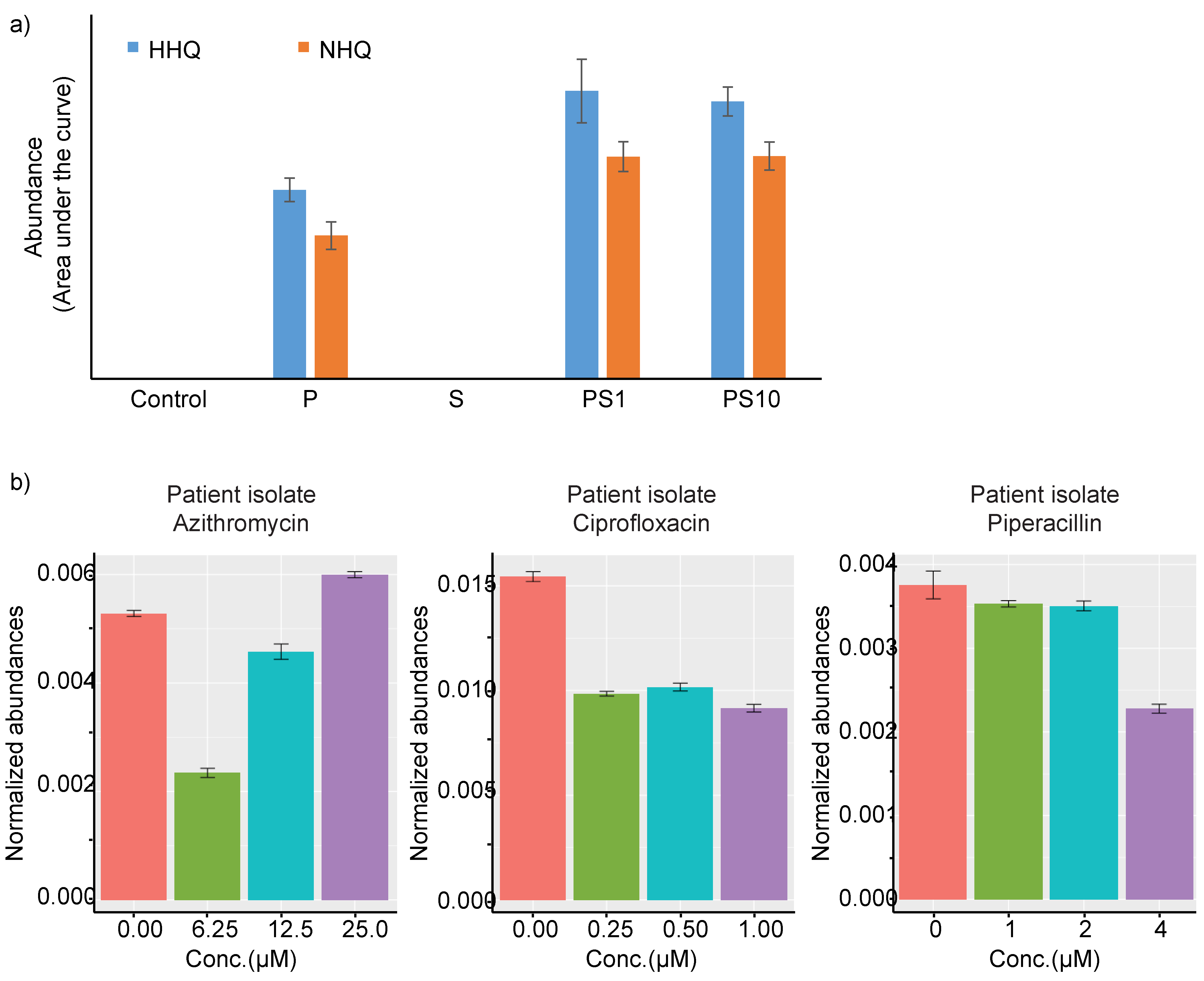

Supplement: FIG S9 [file mSystems.00375-19-sf009.tif]
